# Supplementary material for: Observation of current-induced bulk magnetization in elemental tellurium
Source: Nat Commun. 2017 Oct 16;8:954. doi: 10.1038/s41467-017-01093-3 (PMC5738396; doi:10.1038/s41467-017-01093-3)
Supplement: Supplementary file 1 — Supplementary Information [file 41467_2017_1093_MOESM1_ESM.pdf]

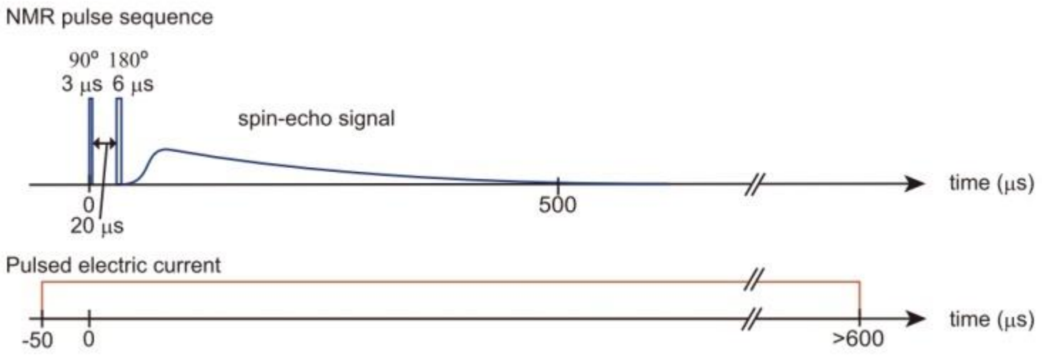

Supplementary Figure 1| Pulse sequence during spin-echo NMR measurements. The NMR pulse sequence was synchronized with a pulsed electric current.

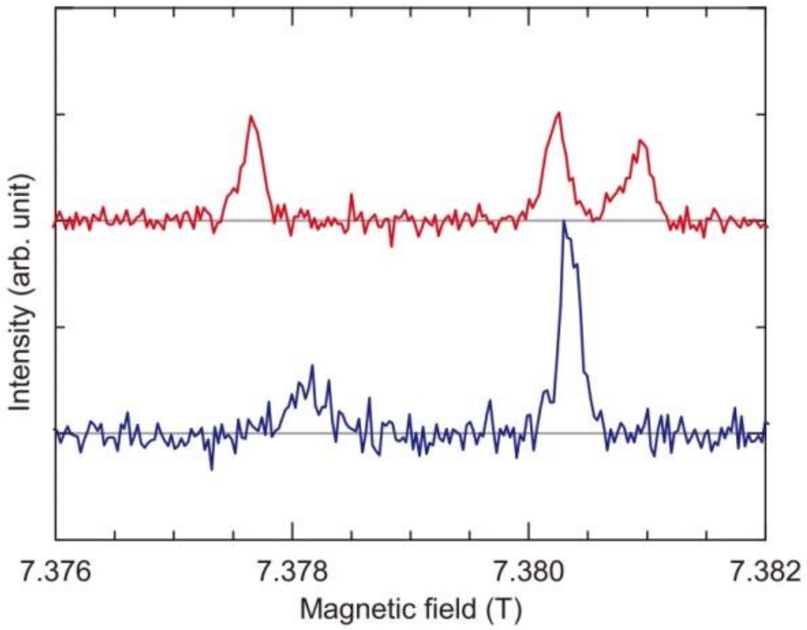

Supplementary Figure 2| ^{125}Te-NMR spectra of single crystal of trigonal tellurium. A magnetic field of 7.3858 T was applied in direction away from the *c* axis (red) and almost parallel to the *c* axis (blue). The latter is the same spectrum as in Fig. 1e.

Supplementary Note 1: Estimation of the current-induced magnetization

In principle, one can estimate the amount of the current-induced magnetization from the current-induced NMR shift observed, using the value of the hyperfine coupling of ^{125}Te in elemental trigonal tellurium. However, at present, it is impossible to estimate the amount of the induced magnetization because there is no reliable source of information on the hyperfine coupling in elemental trigonal tellurium. In other words, reliable K (Knight shift)–χ (spin susceptibility) analyses have not been achieved, despite the long length of time of the elemental tellurium study. This is because elemental trigonal tellurium is a semiconductor with a band gap E_g of ~330 meV, which is much larger than room temperature, and thus the spin susceptibility is too small to be detected. Indeed, the total magnetic susceptibility does not show a temperature dependence in non-doped pure cyrstals below room temperature[1,2]; the experimental value of the spin susceptibily is, therefore, not available.

Nevertheless, we will provide a rough and tentative estimate of the current-induced magnetization. In Supplementary Ref. 3, the Knight shift data of the intrinsic region (350–700 K) is reported. We estimated the hyperfine coupling to be 5.4×10^3 T μ_B^{-1} (where μ_B is the Bohr magneton) by using the reported Knight shift data and theoretically calculated spin susceptibility; the details are provided below.

We calculate the high-temperature spin susceptibility under a magnetic field along the c axis, according to the procedure discussed in Supplementary Ref. 3. First, we adopt the approximate form of the spin susceptibility of trigonal tellurium caused by non-degenarate electrons and holes thermally excited across the band gap: $\chi_s = [\mu_B{}^2 n_e(T) + (2\mu_B)^2 n_h(T)] / k_B T$, where $n_e(T)$ and $n_h(T)$ are the density of the electrons and holes, respectively. Next, we assume the density of the electrons and holes to be $n_e(T) = n_h(T) = (2/h^3)(2\pi k_B T)^{3/2}(m_e^* m_h^*)^{3/4} e^{-E_g/2k_B T}$, where h is the Planck constant, and m_e* and m_h* are the density-of-states effective mass of the conduction and valence bands, respectively. By substituting m_e* = $0.091m_0$ and m_h* = $0.143m_0$ (where m_0 is the free electron mass)[4], we obtain $\chi_s = \mu_B \times [7.50 \times 10^{14}$ (cm^{-3} T^{-1} K$^{-1/2}$)] $\times [T(\mathrm{K})]^{1/2} \times \exp(-E_g / 2k_B T)$. Lastly, we compare this susceptibility with the reported Knight shift, $K = [1.38 \times 10^{-4}$ (K$^{-1/2}$)] $\times [T(\mathrm{K})]^{1/2} \times \exp(-E_g / 2k_B T)$; this allows us to obtain a hyperfine coupling of 5.4×10^3 T μ_B^{-1}. We note that this value does not represent the hyperfine coupling of the uppermost valence band, but rather it is the average value of those of the uppermost valence band and the conduction bands, which can be different. In addition, we neglect the effect of the temperature dependence of the orbital magnetism, which

can also contribute to the temperature dependence of the Knight shift. As a result, the estimated hyperfine coupling is not very reliable.

Nevertheless, if we adopt this hyperfine coupling value (*i.e.*, 5.4 × 10^3 T μ_B^{-1}), the spectral shift of ~0.7 Gauss (under 82 A cm^{-2}) observed in the present study yields a magnetization of 1.3 × 10^{-8} μ_B per site.

Next, we try to compare the above rough estimation of the current-induced magnetization with a theoretical calculation. When an electric field is applied along the *c* (*z*) axis, a current-induced spin polarization $\langle s_z \rangle$ and an electric current density $\langle j_z \rangle$ can be expressed by a Boltzmann transport equation approach in the relaxation-time approximation[5]:

$$\langle s_z \rangle = \frac{3}{2} V \int_{BZ} \frac{d\mathbf{k}}{(2\pi)^3} s_z^h(\mathbf{k}) v_z^h(\mathbf{k}) e E_z \tau \left(\frac{\partial f_0^h}{\partial \varepsilon} \right)_{\varepsilon = E^h(\mathbf{k})},$$

$$\langle j_z \rangle = -e \int_{BZ} \frac{d\mathbf{k}}{(2\pi)^3} \left(v_z^h(\mathbf{k}) \right)^2 e E \tau \left(\frac{\partial f_0^h}{\partial \varepsilon} \right)_{\varepsilon = E^h(\mathbf{k})},$$

where V is a system volume, e (>0) is the elementary charge, $\mathbf{k}$ is the wave vector of a hole, $s^h_z(\mathbf{k})$ is the z-axis component of the spin of a hole at $\mathbf{k}$, $v^h_z(\mathbf{k})$ is the z-axis component of the group velocity of a hole at $\mathbf{k}$, E_z is the z-axis component of an electric field, τ is the scattering time, $E^h(\mathbf{k})$ is the energy dispersion of a hole, $(\partial f^h_0/\partial \varepsilon)_{\varepsilon=E^h(\mathbf{k})}$ is a derivative of the equilibrium distribution function of the holes at $E^h(\mathbf{k})$ with respect to an energy, and BZ denotes the first Brillouin zone. If the energy dependence of τ is neglected, then the following equation is obtained:

$$\langle s_z \rangle = -\frac{3}{2} V \frac{\int_{BZ} d\mathbf{k} s_z^h(\mathbf{k}) v_z^h(\mathbf{k}) \left(\frac{\partial f_0^h}{\partial \varepsilon} \right)_{\varepsilon = E^h(\mathbf{k})}}{e \int_{BZ} d\mathbf{k} \left(v_z^h(\mathbf{k}) \right)^2 \left(\frac{\partial f_0^h}{\partial \varepsilon} \right)_{\varepsilon = E^h(\mathbf{k})}} \langle j_z \rangle .$$

Note that the value of $eE\tau$ is absent in this equation. Below, we use the actual chemical potential at 100 K, $\langle j_z \rangle$ = 82 A cm^{-2},

$$E^h(\mathbf{k}) = -E(-\mathbf{k}) = -[A(k_x^2 + k_y^2) + Bk_z^2 + \sqrt{S^2 k_z^2 + \Delta^2} - \Delta - E_0],$$

$$s_z^h(\mathbf{k}) = -s_z(-\mathbf{k}) = (3/2) \times S k_z \Big/ \sqrt{S^2 k_z^2 + \Delta^2}$$ and

$v_z^{\rm h}(\mathbf{k}) = -\left(2Bk_z + S^2k_z \big/ \sqrt{S^2k_z^2 + \Delta^2}\right)\big/\hbar$, where $\mathbf{k} = (k_x, k_y, k_z)$ is a wave vector from the H or H' points, $A = -32.6$ eV Å^2, $B = -36.4$ eV Å^2, $S = -2.47$ eV Å (for $P3_121$), $\Delta = 63$ meV, and $E_0 = 2.4$ meV. As a result, the density of the current-induced spin polarization is calculated to be $\langle s_z\rangle/V = -4.1 \times 10^{13}$ cm^{-3}. The current-induced magnetization per tellurium atom $M_{\rm atom}$ is calculated to be $M_{\rm atom} = g_{\rm J=3/2}\mu_{\rm B}\langle s_z\rangle \times V_{\rm atom} \sim 1.9 \times 10^{-9}\ \mu_{\rm B}$, where $g_{\rm J=3/2} = -4/3$ is the Landé g-factor of $J = 3/2$ ($S = 1/2$, $L = 1$), and $V_{\rm atom} = 34$ Å^3 is the atomic volume of tellurium. This theoretically calculated result, $\sim 10^{-9}\ \mu_{\rm B}$ per site, is comparable to the above estimation obtained from the NMR shift, even though both the estimations are rough. Note that the present calculation quantitatively reproduces the result of a similar calculation in Supplementary Ref. 6 with accuracy less than a few per cents, considering the different conditions between the two studies, such as temperature, carrier densities, and band parameters.

In spite of the rough agreement between the experimental estimation and the theoretical calculation, we emphasize again that the hyperfine coupling value described above is not very reliable and may contain considerable uncertainties. Further quantitative discussions would therefore be needed with regards to current-induced bulk magnetization.

Supplementary References

1. Fukuroi, T. & Yasuhara, K On the Magnetic Susceptibility of Tellurium and the Magnetic Anisotropy of its Single Crystal. *J. Jap. Inst. Metals* **19,** 118–122 (1955).

2. Fischer, G. & Hedgcock, F. T. Magnetic susceptibility and galvanomagnetic effects in pure and P-type tellurium. *J. Phys. Chern. Solids* **17,** 246–253 (1961).

3. Selbach, H., Kanert, O. & Wolf, D. NMR investigation of the diffusion and conduction properties of the semiconductor tellurium. I. Electronic properties. *Phys. Rev. B* **19,** 4435–4443 (1979).

4. Shinno, H., Yoshizaki, R., Tanaka, S., Doi, T. & Kamimura, H. Conduction Band Structure of Tellurium. *J. Phys. Soc. Japan* **35,** 525–533 (1973).

5. Li, P. & Appelbaum, I. Interpreting current-induced spin polarization in topological insulator surface states. *Phys. Rev. B* **93,** 220404(R) (2016).

6. Shalygin, V. A., Sofronov, A. N., Vorob'ev, L. E. & Farbshtein, I. I. Current-Induced Spin Polarization of Holes in Tellurium. *Phys. Solid State* **54,** 2362–2373 (2012).
